# Zoonotic cutaneous leishmaniasis caused by *Leishmania major*: Do humans play a role in amplifying transmission?

**DOI:** 10.1051/parasite/2026030

**Published:** 2026-05-25

**Authors:** Karim Aoun, Fida Maatallah, Aïda Bouratbine

**Affiliations:** 1 Laboratory of Epidemiology and Ecology of Parasites, Institut Pasteur de Tunis 1002 Tunis Tunisia; 2 Research Laboratory 16-IPT-06: Medical Parasitology, Biotechnology and Biomolecules, Institut Pasteur de Tunis, University of Tunis-El Manar Tunis Tunisia; 3 Department of Parasitology, Institut Pasteur de Tunis 1002 Tunis Tunisia

**Keywords:** Zoonotic Cutaneous Leishmaniasis, *Leishmania major*, *Phlebotomus papatasi*, Human, Transmission

## Abstract

Zoonotic cutaneous leishmaniasis (ZCL) caused by *Leishmania* (*L.*) *major* is the most widespread form of CL worldwide, with hundreds of thousands of cases annually. It is associated with significant morbidity and economic burden, particularly in North Africa and the Middle East. For over a century, the zoonotic transmission of ZCL, involving wild gerbils (Muridae: Gerbillinae) as the reservoir and *Phlebotomus* (*Ph*.) *papatasi* as the vector, has been considered an established fact, with no contradictory debate. However, the pronounced endophilic and anthropophilic behaviors of *Ph. papatasi*, the exposure of multiple and large lesions, although with relatively limited persistence, to sandflies during their seasonal activity, and the simultaneous detection of human and *L. major* DNA within the vector, suggest a contribution of humans to the parasite cycle. Conversely, the high incidence of the disease in anthropized foci and the high prevalence of family cases highlight the role of humans in amplifying transmission. The experiments carried out by Adler and Theodor in the 1920s in Palestine and the more recent studies done by Fatemi *et al*. (2018) in Iran that identified *L. major* infectious metacyclic forms in *Ph. papatasi* fed on ZCL lesions reinforce this hypothesis. Additional xenodiagnoses-based approaches could provide definitive answers and lead to better management and control of ZCL, leading to a recommendation of updated preventive measures at the household level. This review aims to report on what is currently known about the zoonotic transmission of *L. major*, and to develop arguments supporting the hypothesis that humans play a potential role as “reservoir host” of the parasite, albeit a complementary one.

## Abstract (fr)

La leishmaniose cutanée zoonotique (LCZ) due à *Leishmania (L.) major* est la forme de leishmaniose cutanée la plus répandue au monde, avec des centaines de milliers de cas par an. Elle est associée à une morbidité et un fardeau économique importants, notamment en Afrique du Nord et au Moyen-Orient. Pendant plus d’un siècle, la transmission zoonotique de la LCZ, impliquant la gerbille sauvage (Muridae : Gerbillinae) comme réservoir et *Phlebotomus (Ph.) papatasi* comme vecteur, a été considérée comme un fait établi, sans controverse. Cependant, les comportements endophiles et anthropophiles marqués de *Ph. papatasi*, l’exposition de lésions multiples et étendues, bien que de persistance relativement limitée, aux phlébotomes lors de leur activité saisonnière, et la détection simultanée d’ADN humain et de *L. major* chez le vecteur, suggèrent une contribution des humains au cycle parasitaire. D'autre part, la forte incidence de la maladie dans les foyers anthropisés et la prévalence élevée de cas familiaux soulignent le rôle des humains dans l'amplification de la transmission. Les expériences d'Adler et Theodor dans les années 1920 en Palestine et l'étude plus récente de Fatemi *et al*. (2018) en Iran, qui ont identifié des formes métacycliques infectieuses de *L. major* chez *Ph. papatasi* nourri de lésions de la LCZ, renforcent cette hypothèse. Des approches complémentaires basées sur le xénodiagnostic pourraient apporter des réponses définitives et permettre une meilleure prise en charge et un meilleur contrôle de la LCZ, ce qui pourrait conduire à recommander des mesures préventives actualisées au niveau des ménages. Cette revue vise à présenter l'état actuel des connaissances sur la transmission zoonotique de *L. major* et à développer des arguments suggérant que les humains pourrait jouer un rôle de réservoir pour le parasite, bien que complémentaire.

## Introduction

Cutaneous leishmaniasis (CL) is one of the most common vector-borne diseases worldwide with approximately 600,000 to 1,000,000 new cases per year [10, 31, 84]. It is currently endemic in 89 countries where constantly increasing incidence and morbidity have been observed over the past 40 years due to environmental changes favorable to transmission and spread [14, 84]. The Middle East (ME), North Africa (NA), Central Asia, and South America [10, 14, 84], and more recently West Africa [41], are the most affected regions with a high incidence of cases exceeding 1 per 100 inhabitants in some areas [14]. Unfortunately, CL remains a neglected disease that primarily affects regions and countries with limited resources and scarce means for management and control [84]. In the Old World, *Leishmania* (*L*.) *major* (Yakimoff and Schokhor, 1914) and *L. tropica* (Wright, 1903) are by far the most prevalent causative species [10, 14, 84]. Transmission of *L. major* is considered rural and zoonotic, while transmission of *L. tropica* is mainly described as urban and anthroponotic [14, 72], although recent findings have suggested the possible involvement of dogs [7]. *Leishmania major* is associated with the highest incidence and the widest geographical distribution [10, 14, 84]. It is the main species involved in *Leishmania* transmission in the Maghreb countries (NA) where zoonotic CL (ZCL) is considered to be a major health threat [14, 25].

The first descriptions of ZCL in NA were reported towards the end of the 19th century by Hamel and Dépéret and Boinet who documented regional cases in the oases of Biskra (Algeria) and Gafsa (Tunisia) in 1860 and 1884, respectively [46]. Since then, the number of cases has remained relatively stable and low, until sudden outbreaks occurred in the 1980s [14, 23]. ZCL then spread rapidly through the central and southern regions of all the Maghreb countries [14, 25, 56, 75]. This was likely favored by the construction of dams and hill lakes and the promotion of irrigation and agriculture, which may have contributed to increased densities of reservoir hosts and vectors [21, 22, 24, 51]. Moreover, concomitant, rapid, and uncontrolled urbanization have likely led to increased human exposure to the parasite [14, 77]. Currently, ZCL is the cause of significant physical and psychological morbidity as well as considerable economic losses [14, 33, 72, 84]. Its incidence exceeds thousands of cases per year in Algeria, Morocco, Libya, and Tunisia, with a peak that reached 25,000 cases in Algeria in 2005 [14, 25].

Despite its high incidence and morbidity and its tendency to emerge and expand [14, 25, 84], relatively few studies have focused on ZCL’s risk factors. Studies depicting the modalities of parasite circulation, particularly those concerning the precise roles played by hosts and vectors, remain rare. The same applies to studies on prevention and control options. The historic eco-epidemiologic knowledge developed in the first half of the 20th century, a period when case incidence was significantly lower, is often treated as unquestionable “dogma” and is still cited in most publications. Therefore, it is widely assumed that *L. major* is a zoonotic parasite whose reservoirs are exclusively wild rodents belonging to the Gerbillinae subfamily [5, 18, 20, 50, 53, 73], and that its life cycle occurs in suitable biotopes between gerbils and the sandfly vectors *Phlebotomus (Ph.) papatasi* in NA and the ME [24, 45, 76, 79, 85], and *Ph. duboscqi* in West Africa [41]. Humans are considered accidental and dead-end hosts contracting the infection through the bite of a sandfly that has acquired the parasite from a wild rodent reservoir [64]. However, besides the principal role of rodents as reservoirs, several entomological data as well as recent eco-epidemiologic observations argue in favor of a secondary or complementary role of humans. The reports presented by Adler and Theodor in the 1920s [3, 4], and recently the studies carried out by Fatemi *et al.* [49], can also be interpreted as experimental proof of this hypothesis. The objective of this review was to discuss the “paradigm” of the strict zoonotic nature of ZCL and to question a potential “reservoir role” of humans.

## Current knowledge on the components and dynamics of the *L. major* transmission cycle

### Phlebotomus papatasi

*Phlebotomus papatasi* (Scopoli, 1786) is the confirmed vector of *L. major* in NA and the ME and the first sandfly species described of the 1,026 sandfly species reported to date [24, 52, 56, 58, 63, 79, 82, 85]. It is widely distributed across the Old World, extending from East to West from Morocco to Central Asia, passing through the Maghreb, the Mediterranean Basin, and the ME countries [6, 45, 48, 65], where it is abundant in semi-arid and arid zones [24, 42, 55]. In the Maghreb and ME, *Ph. papatasi* is seasonally active from May to October [37, 43, 55, 62], and even November in some warmer regions [38]; a diapause at the fourth larval stage allows it to survive the cold winter months [71]. Only females are hematophagous and bite during the evening and early morning [42, 76]. It is noteworthy that surveys have shown high density and abundance of *Ph. papatasi* in ZCL endemic areas and a strong correlation with disease transmission [19, 25, 35, 55, 63].

### Rodent reservoirs

The reservoir hosts of *L. major* are wild rodents belonging to the Gerbillinae subfamily [14, 72]. Dogs have occasionally been reported as infected by *L. major*, although without any evident role in transmission [8, 66]. Depending on the geographic region, the following genera of gerbils are involved: *Psammomys*, *Meriones*, *Rhombomys*, and *Mastomys* [5, 20, 26, 41, 47, 50, 53, 73].

*Psammomys obesus* (Cretzschmar, 1828), also known as the fat sand rat, is the main reservoir host of *L. major* in NA and the ME [18, 20, 26, 51, 53, 58]. It is particularly abundant in semi-desert areas, especially in the chott and sabkha regions, where halophytic vegetation (chenopods) grows abundantly and constitutes its almost exclusive food source [26, 44, 50]. *Psammomys* rodents are diurnal and relatively sedentary. Their burrows provide highly favorable conditions for sandfly survival and reproduction, offering darkness, coolness, humidity, organic matter, and blood meals [51, 81].

The *Meriones* gerbils, *Meriones (M.) shawi* (Duvernoy, 1842) and *M. libycus* (Lichtenstein, 1823) are known to be reservoirs of *L. major* in NA and the ME, respectively [47, 53, 70, 73, 74]. They are granivorous and inhabit agricultural fields, particularly wheat and corn, as well as vegetable plots. *Meriones* burrows are typically found at the edges of fields, often on raised earth or clay embankments “touabis” that separate them. They are also commonly encountered along riverbanks and dry wadis, in oases and, increasingly, in peri-domestic areas, where they inhabit animal shelters, abandoned constructions, and even dwellings [20, 49]; these sites also provide favorable habitats for *Phlebotomus* [51, 55, 76].

*Rhombomys opimus* (Lichtenstein, 1823) and *Mastomys natalensis* (Smith, 1834) are also murids closely related to *Meriones* and serve as reservoirs for *L. major* in Central Asia and West Africa, respectively [5, 41].

Depending on the region, all these gerbils are highly abundant in active ZCL foci, with *Leishmania* infection rates sometimes exceeding 50% [5, 50]. Infections mainly affect their ears and nose but do not appear to impair their vitality [26, 47, 50, 51]. Additionally, nearly 30% of infected gerbils remain clinically asymptomatic yet are still capable of transmitting the parasite to sandflies [5, 26, 50]. Their ability to survive for over a year makes them ideal reservoirs for maintaining the parasite during the winter, when sandflies are inactive and transmission ceases.

### Key components of the *L. major* epidemiological cycle

It has been widely accepted that *L. major* circulates in the wild between its vector, *Ph. papatasi*, and rodent reservoirs. Transmission primarily occurs inside rodent burrows, but also around animal shelters and rural dwellings [2, 14, 53, 73]. Humans are considered accidental hosts and acquire the infection through the bite of an infected female sandfly. This is made possible by the proximity of humans to rodent and *Ph. papatasi* habitats. The high prevalence of ZCL in hyperendemic foci is therefore explained by the high densities of both sandflies and rodents in these areas [35, 50, 53, 55].

The rodent species *P. obesus* is traditionally responsible for most parasite circulation, particularly in and around chotts and sabkhas [14, 18, 50]. It is thought to play a key role in amplifying local parasite transmission [51]. This was evident during the ZCL epidemics in Algeria and Tunisia in the 1980s. The epidemics occurred around the Chott Hodna in Biskra and M’sila, Algeria, and the sabkhas of central Tunisia, particularly in Kairouan and Sidi Bouzid, before spreading to other parts of the two countries [14, 25, 26]. Conversely, the *Meriones* species, which are more prevalent in agricultural and peri-domestic areas, are believed to be responsible for spreading the parasite and disseminating it into new areas, thanks to their low dietary requirements and greater mobility [14, 51, 54].

In addition to ecological changes, the ZCL epidemic in the 1980s was also the result of social and behavioral factors that have significantly increased human exposure to the parasite. Rapid population growth and uncontrolled urbanization led to higher population densities and to the encroachment of human settlements into areas of high *L. major* transmission, particularly around chotts, sabkhas, and oases [14, 25, 49]. Furthermore, the intensive use of water resources, coupled with the promotion of irrigation and agriculture in arid and even desert regions, has led to migration of susceptible non-immunized agricultural workers and their families to these areas alongside a proliferation of sandflies and *Meriones* [14, 22, 34].

## Arguments supporting the potential role of humans in *L. major* transmission

### Behavior of *Ph. papatasi*

#### Endophily

*Phlebotomus papatasi* is the most abundant species found inside dwellings in ZCL endemic areas [35, 55, 78]. It is recognized as a peri-domestic species exhibiting strong endophilic behavior [1, 42]. It thrives in anthropized rural environments where females find optimal conditions and all the resources needed during their lifespan [1, 2, 42, 55, 63]. Poorly maintained dwellings with cracks in walls, crevices, and hay deposits provide suitable resting places and breeding sites for gravid females [2, 32, 37, 78, 81]. Furthermore, sandflies are weak fliers and are usually sedentary, staying not far from their breeding sites, which explains their close association with human dwellings [76]. This aligns with the fact that ZCL is mainly attributed to indoor transmission, particularly given that *Ph. papatasi* bite at the end of the night when most inhabitants are indoors and asleep [42].

#### Anthropophily

Although sandflies are essentially opportunistic and their feeding preferences are conditioned by their habitat and blood source availability [76], the attraction of *Ph. papatasi* to humans, particularly in peri-domestic environments has been well documented [1, 37, 42, 55]. Most surveys in ZCL endemic areas reported that blood meals from captured females are mostly of human origin, sometimes exceeding 70% of all blood sources [19, 61, 68, 85].

Both endophily and anthropophily of *Ph. papatasi* point to the high probability of this species becoming infected through an infective human bite and raise the question of the possibility of human-to-human transmission.

### Epidemiologic features of ZCL

#### Rapid and sudden increase in incidence

The relatively recent increase in ZCL incidence has been attributed by almost all authors, quite rightly, to environmental changes that have brought humans into closer contact with the vector and that have exposed them to the zoonotic cycle of *L. major*. The sudden onset and extensive epidemic scale of this increase, from a few dozen cases to thousands in some regions [14, 25, 84], are reminiscent of vector-borne diseases that involve humans as reservoirs, such as malaria, African trypanosomiasis, and dengue fever [57]. This observation suggests that the potential role of humans in amplifying transmission as secondary reservoirs should be considered in epidemiologic assessments.

#### Frequency of cases among household members and close contacts

Several studies have reported a higher prevalence of ZCL cases among household members. In their respective studies in Gafsa, Tunisia, and M’sila, Algeria, Bellali and Benikhlef found that around half of the cases occurred within family clusters [21, 25]. Some authors have explicitly identified the family environment as a risk factor for the disease [21, 59]. Bellali’s study in Gafsa, as well as certain WHO reports, have also suggested a positive correlation between ZCL incidence and household occupancy. This makes overcrowding another indoor risk factor for ZCL [21, 60, 84]. Although household members of confirmed cases are exposed to the same environmental conditions (infected sandflies and rodents in the area), and therefore share similar exposure risks, these findings highlight the fact that infected individuals may play a role in amplifying parasite transmission.

#### Higher ZCL incidence among children and women

Most studies show that children and women are the most affected by ZCL [25, 27, 40]. These incidence rates could suggest intra-domiciliary contamination involving humans in amplifying transmission. Of note, in socially traditional and conservative rural areas, women as well as children under five years of age rarely leave their homes in the evening when sandflies are active. The location of lesions on women’s limbs also supports the indoor transmission hypothesis since, for social reasons, their arms and legs are usually covered outdoors [13, 59]. Overall, it is worth noting that the high incidence among children is also related to their lower immunity to the parasite, which is gradually acquired with age [34].

#### ZCL outbreaks without identified rodent reservoirs

ZCL cases in NA and the ME are typically recorded in areas where *Meriones* and/or *P. obesus* are nearby. However, some outbreaks, sometimes quite active, such as those in Bordj Bou Arreridj in northern Algeria and Métlaoui in central-western Tunisia [12, 14, 25], do not offer these conditions and the parasite reservoir remains hypothetical [49]. Therefore, the rather urban outbreak in Métlaoui, where dozens of indigenous cases of ZCL are reported annually, has always raised questions. The involvement of synanthropic rodents as a secondary reservoir, such as *Ctenodactylus gondii*, even though its biotopes are quite far from the city, or *Rattus rattus*, the only rodent in the vicinity, has been suggested as they have been reported to be mildly infected with *L. major* [28, 80]. In such situations, the possibility of a human role in amplifying the transmission and acting as a “secondary reservoir” created locally from imported cases from neighboring endemic villages cannot be entirely ruled out. However, the possibility of the epidemiologic cycle being maintained solely by humans in the absence of rodent animal reservoirs remains questionable.

### Clinical features of ZCL lesions

#### Short incubation period and sufficient duration of ZCL lesions

The incubation period of ZCL is the shortest of all CL forms prevalent in the Maghreb [13, 16]. It has recently been estimated to average 5 weeks in Tunisia, sometimes with a shorter duration of 2–3 weeks [15, 16]. Thus, following the peak density of *Ph. papatasi* in August [37, 43, 55], the first lesions appear in September [13, 16, 25]. These highly inflammatory and parasitized lesions could be potentially infectious to sandflies [17, 49]. Before self-healing, they persist for several months (average of 2 to 6 months) and coexist in September, October, and even November with sill active sandflies [14, 30, 35, 37, 39, 55, 72]. This provides sufficient opportunities to females to become infected from CL lesions and subsequently transmit mature parasites to other household members [49].

#### High exposure of ZCL lesions to sandflies

In more than 60–70% of cases, ZCL lesions are multiple, with an average of nearly three lesions per patient [9, 13, 25, 39, 59]. These lesions are often large and located on limbs which are frequently uncovered and exposed during the warm seasons when transmission occurs [11, 13]. The combination of these three lesion characteristics (number, size, and location) results in large, parasitized areas that are easily accessible to *Ph. papatasi*. In addition, several authors have reported that hosts infected with *Leishmania* are particularly attractive to sandflies, thereby amplifying transmission [36, 67, 69]. Although such reports concerned systemic leishmaniasis in dogs, these findings could question interactions between parasitized mammals and *Ph. papatasi* females.

### Simultaneous detection of human blood and *L. major* DNA in *Ph. papatasi* and experimental evidence of human-to-human transmission

Several reports have detected both human blood and *L. major* DNA within the same *Ph. papatasi* female, which suggests that females of the species can feed on ZCL lesions and acquire the parasites [19]. This was recently demonstrated by Fatemi *et al.* in Iran, who detected, despite a low infectiousness rate (2 infected females out of 93 blood-fed), metacyclic promastigotes following a xenodiagnoses experiment on a ZCL lesion [49]. In this context, the experiments conducted by Adler and Theodor in Jericho in the 1920s are particularly noteworthy [3, 4]. They demonstrated vector-mediated human-to-human transmission of *L. major* via *Ph. papatasi*. Although the authors used “*L. tropica”* to refer to the species of the parasite in question, this is likely to correspond to *L. major*. The same misbelief was adopted by the Sergent brothers in Biskra, Algeria, the historical and exclusive focus of *L. major* [83]. This taxonomic confusion persisted until 1973, when Bray formally differentiated the two subspecies, establishing *L. major* and *L. tropica* as distinct species [29]. Therefore, the results of Adler and Theodor that were recently sustained by Fatemi *et al.* represent a further argument that justifies our questions about the possible role of humans as a source of infection for sandflies.

## Conclusion

The biological cycle of *L. major* is still considered exclusively zoonotic. However, the high exposure of CL lesions, combined with the strongly endophilic and anthropophilic behavior of *Ph. papatasi*, raises legitimate questions regarding the potential role of humans as a secondary “parasite reservoir”. Outbreaks occurring in urban areas, high proportions of family cases, and high incidence rates among children and women also suggest amplified local transmission in households, independently of rodent involvement. Moreover, the experiments by Adler and Theodor and Fatimi *et al*. provided evidence of potential anthroponotic parasite circulation. Nonetheless, this hypothesis has never been debated, investigated, or ruled out, highlighting the need for targeted research into *L. major* transmission. It is noteworthy that elucidating the role of humans in parasite transmission would enable a reassessment of the risk factors for the disease and lead to an update of the current recommendations for prevention, management, and control. In addition to medical treatment, simple household measures could be relevant, such as covering lesions and further promoting the use of repellents and bed-nets. These new measures could be beneficial, especially since all programs against ZCL, which rely primarily on rodent control, have proven ineffective.

## References

[1] Abonnenc E. 1959. Comparative study of the females of *Phlebotomus papatasi* Scopoli, *Phlebotomus papatasi var. bergeroti* Parrot & *Phlebotomus duboscqi* Neveu-Lemaire. Archives de l’Institut Pasteur d’Algérie, 37, 329–339. 13670657

[2] Abonnenc E. 1972. Les phlébotomes de la région éthiopienne (Diptera, Psychodidae). Paris: ORSTOM.

[3] Adler S, Ber M. 1941. Transmission of *Leishmania tropica* by the bite of *Phlebotomus papatasii*. Nature, 148, 227–227.

[4] Adler S, Theodor O. 1925. The experimental transmission of cutaneous leishmaniasis to man from *Phlebotomus papatasii*. Annals of Tropical Medicine and Parasitology, 19, 365–371.

[5] Akhavan AA, Yaghoobi-Ershadi MR, Khamesipour A, Mirhendi H, Alimohammadian MH, Rassi Y, Arandian MH, Jafari R, Abdoli H, Shareghi N, Ghanei M, Jalali-zand N. 2010. Dynamics of *Leishmania* infection rates in *Rhombomys opimus* (Rodentia: Gerbillinae) population of an endemic focus of zoonotic cutaneous leishmaniasis in Iran. Bulletin de la Société de Pathologie Exotique, 103, 84–89. 20390397 10.1007/s13149-010-0044-1

[6] Akhoundi M, Kuhls K, Cannet A, Votýpka J, Marty P, Delaunay P, Sereno D. 2016. A historical overview of the classification, evolution, and dispersion of *Leishmania* parasites and sandflies. PLoS Neglected Tropical Diseases, 10, e0004349. 26937644 10.1371/journal.pntd.0004349 PMC4777430

[7] Akhtardanesh B, Sadr S, Khedri J, Bamorovat M, Salarkia E, Sharifi I. 2024. Canine leishmaniasis caused by *Leishmania tropica* in southeastern Iran: a case series study. Scientific Reports, 14, 25599. 39465272 10.1038/s41598-024-76301-4 PMC11514237

[8] Alanazi AD, Rahi AA, Ali MA, Alyousif MS, Alanazi IO, Mahmoud MS, Abdel-Shafy S, Alraey YA, Alouffi AS. 2019. Molecular detection and phylogenetic analysis of *Leishmania major* in stray dogs in Riyadh Province, Saudi Arabia. Tropical Biomedicine, 36, 315–323. 33597392

[9] Al-Jawabreh A, Schoenian G, Hamarsheh O, Presber W. 2006. Clinical diagnosis of cutaneous leishmaniasis: A comparison study between standardized graded direct microscopy and ITS1-PCR of Giemsa-stained smears. Acta Tropica, 99, 55–61. 16920056 10.1016/j.actatropica.2006.07.001

[10] Alvar J, Vélez ID, Bern C, Herrero M, Desjeux P, Cano J, Jannin J, Boer M den. 2012. Leishmaniasis worldwide and global estimates of its incidence. PLoS ONE, 7, e35671. 22693548 10.1371/journal.pone.0035671 PMC3365071

[11] Amiri Z, Moradi A, Farkhani EM. 2024. Predictors of lesion size among people with cutaneous leishmaniasis (2016-2021): A multilevel analysis. Iranian Biomedical Journal, 28, 67.

[12] Aoun K. 2008. Épidémiologie de *Leishmania* (*L*.) *infantum*, *L. major* et *L. killicki* en Tunisie: Résultats et analyse de l’identification de 226 isolats humains et canins et revue de la littérature. Bulletin de la Société de Pathologie Exotique, 101, 323. 18956815 10.3185/pathexo3201

[13] Aoun K, Ben Abda I, Bousslimi N, Bettaieb J, Siala E, Ben Abdallah R, Benmously R, Bouratbine A. 2012. Caractérisation comparative des trois formes de leishmaniose cutanée endémiques en Tunisie. Annales de Dermatologie et de Vénéréologie, 139, 452–458. 22721477 10.1016/j.annder.2012.04.154

[14] Aoun K, Bouratbine A. 2014. Cutaneous leishmaniasis in North Africa: a review. Parasite, 21, 14. 24626301 10.1051/parasite/2014014 PMC3952656

[15] Aoun K, Chetoui A, Rhaiem A, Ghrab J, Dani R, Bouratbine A. 2005. Early cutaneous leishmaniasis in a 7-day-old newborn. Médecine Tropicale, 65, 394–395. 16548498

[16] Aoun K, Kalboussi Y, Ben Sghaier I, Souissi O, Hammami H, Bellali H, Bouratbine A. 2020. Assessment of incubation period of cutaneous leishmaniasis due to *Leishmania major* in Tunisia. American Journal of Tropical Medicine and Hygiene, 103, 1934–1937. 32901597 10.4269/ajtmh.20-0439 PMC7646806

[17] Aoun K, Tebrouri M, Ben Abdallah R, Bellali H, Souissi O, Bouratbine A. 2020. Contribution de la PCR en temps réel dans le diagnostic biologique de la leishmaniose cutanée : expérience de l’institut Pasteur de Tunis. Bulletin de la Société de Pathologie Exotique, 113, 251–253. 33881254 10.3166/bspe-2020-0150

[18] Ashford RW, Schnur LF, Chance ML, Samaan SA, Ahmed HN. 1977. Cutaneous leishmaniasis in the Libyan Arab Republic: preliminary ecological findings. Annals of Tropical Medicine and Parasitology, 71, 265–271. 921363 10.1080/00034983.1977.11687190

[19] Azmi K, Schonian G, Abdeen Z. 2020. Specification of blood meals ingested by female sand flies caught in Palestinian foci and identification of their concomitant leishmanial infections. PLoS Neglected Tropical Diseases, 14, e0008748. 33017399 10.1371/journal.pntd.0008748 PMC7561261

[20] Belazzoug S. 1983. Isolation of *Leishmania major* Yakimoff & Schokhor, 1914 from *Psammomys obesus* Gretzschmar, 1828 (Rodentia: Gerbillidae) in Algeria. Transactions of the Royal Society of Tropical Medicine and Hygiene, 77, 876. 10.1016/0035-9203(83)90314-0 6364474

[21] Bellali H, Ben Alaya N, Ahmadi Z, Ennigrou S, Chahed MK. 2015. Eco-environmental, living conditions and farming issues linked to zoonotic cutaneous leishmaniasis transmission in Central Tunisia: a population-based survey. International Journal of Tropical Medicine and Public Health, 5(1), 1.

[22] Bellali H, Chemak F, Nouiri I, Ben Mansour D, Ghrab J, Chahed MK. 2017. Zoonotic cutaneous leishmaniasis prevalence among farmers in Central Tunisia, 2014. Journal of Agromedicine, 22(3), 244–250. 28402250 10.1080/1059924X.2017.1318725

[23] Ben Ismail R, Gradoni L, Gramiccia M, Bettini S, Ben Rachid MS, Garraoui A. 1986. Epidemic cutaneous leishmaniasis in Tunisia: biochemical characterization of parasites. Transactions of the Royal Society of Tropical Medicine and Hygiene, 80, 669–670. 3810806 10.1016/0035-9203(86)90175-6

[24] Benallal KE, Garni R, Harrat Z, Volf P, Dvorak V. 2022. Phlebotomine sand flies (Diptera: Psychodidae) of the Maghreb region: A systematic review of distribution, morphology, and role in the transmission of the pathogens. PLoS Neglected Tropical Diseases, 16, e0009952. 34990451 10.1371/journal.pntd.0009952 PMC8735671

[25] Benikhlef R, Aoun K, Boudrissa A, Ben Abid M, Cherif K, Aissi W, Benrekta S, Boubidi SC, Späth GF, Bouratbine A, Sereno D, Harrat Z. 2021. Cutaneous leishmaniasis in Algeria; highlight on the focus of M’Sila. Microorganisms, 9, 962. 33947003 10.3390/microorganisms9050962 PMC8146893

[26] Ben-Ismail R, Ben Rachid MS, Gradoni L, Gramiccia M, Helal H, Bach-Hamba D. 1987. Zoonotic cutaneous leishmaniasis in Tunisia: study of the disease reservoir in the Douara area. Annales de la Société Belge de Médecine Tropicale, 67, 335–343. 3447519

[27] Bousslimi N, Aoun K, Ben-Abda I, Ben-Alaya-Bouafif N, Raouane M, Bouratbine A. 2010. Epidemiologic and clinical features of cutaneous leishmaniasis in southeastern Tunisia. American Journal of Tropical Medicine and Hygiene, 83, 1034–1039. 21036833 10.4269/ajtmh.2010.10-0234 PMC2963965

[28] Bousslimi N, Ben-Ayed S, Ben-Abda I, Aoun K, Bouratbine A. 2012. Natural infection of North African gundi (*Ctenodactylus gundi*) by *Leishmania tropica* in the focus of cutaneous leishmaniasis, Southeast Tunisia. American Journal of Tropical Medicine and Hygiene, 86, 962–965. 22665601 10.4269/ajtmh.2012.11-0572 PMC3366540

[29] Bray RS, Ashford RW, Bray MA. 1973. The parasite causing cutaneous leishmaniasis in Ethiopia. Transactions of the Royal Society of Tropical Medicine and Hygiene, 67, 345–348. 4778189 10.1016/0035-9203(73)90111-9

[30] Bryceson ADM. 1986. Clinical variations associated with various taxa of *Leishmania*. Applications éco-épidémiologiques (Coll. Int. CNRS/INSERM, 1984). IMEEE: Montpellier. p. 221–228.

[31] Burza S, Croft SL, Boelaert M. 2019. Leishmaniasis – Authors’ reply. Lancet, 393, 872–873. 10.1016/S0140-6736(18)33057-5 30837140

[32] Cecílio P, Cordeiro-da-Silva A, Oliveira F. 2022. Sand flies: Basic information on the vectors of leishmaniasis and their interactions with *Leishmania* parasites. Communications in Biology, 5, 305. 10.1038/s42003-022-03240-z PMC8979968 35379881

[33] Chahed MK, Bellali H, Jemaa SB, Bellaj T. 2016. Psychological and psychosocial consequences of zoonotic cutaneous leishmaniasis among women in Tunisia: Preliminary findings from an exploratory study. PLOS Neglected Tropical Diseases, 10, e0005090. 27788184 10.1371/journal.pntd.0005090 PMC5082956

[34] Chamakh-Ayari R, Chenik M, Chakroun AS, Bahi-Jaber N, Aoun K, Meddeb-Garnaoui A. 2017. *Leishmania major* large RAB GTPase is highly immunogenic in individuals immune to cutaneous and visceral leishmaniasis. Parasites & Vectors, 10, 185. 28416006 10.1186/s13071-017-2127-3 PMC5393016

[35] Chelbi I, Kaabi B, Béjaoui M, Derbali M, Zhioua E. 2009. Spatial correlation between *Phlebotomus papatasi* Scopoli (Diptera: Psychodidae) and incidence of zoonotic cutaneous leishmaniasis in Tunisia. Journal of Medical Entomology, 46, 400–402. 19351095 10.1603/033.046.0229

[36] Chelbi I, Maghraoui K, Zhioua S, Cherni S, Labidi I, Satoskar A, Hamilton JGC, Zhioua E. 2021. Enhanced attraction of sand fly vectors of *Leishmania infantum* to dogs infected with zoonotic visceral leishmaniasis. PLOS Neglected Tropical Diseases, 15, e0009647. 34314425 10.1371/journal.pntd.0009647 PMC8345872

[37] Chelbi I, Mathlouthi O, Zhioua S, Fares W, Boujaama A, Cherni S, Barhoumi W, Dachraoui K, Derbali M, Abbass M, Zhioua E. 2020. The impact of illegal waste sites on the transmission of zoonotic cutaneous leishmaniasis in Central Tunisia. International Journal of Environmental Research and Public Health, 18, 66. 33374115 10.3390/ijerph18010066 PMC7795373

[38] Chelbi I, Zhioua E. 2007. Biology of *Phlebotomus papatasi* (Diptera: Psychodidae) in the laboratory. Journal of Medical Entomology, 44, 597–600. 17695013 10.1603/0022-2585(2007)44[597:boppdp]2.0.co;2

[39] Chiheb S, Slaoui W, Mouttaqui T, Riyad M, Benchikhi H. 2014. Cutaneous leishmaniasis by *Leishmania major* and *Leishmania tropica* in Morocco: comparative epidemioclinical aspects of 268 cases. Pan African Medical Journal, 19, 160. 25810796 10.11604/pamj.2014.19.160.2613 PMC4362619

[40] Chraiet-Rezgani K, Bouafif-Ben Alaya N, Habboul Z, Hajjej Y, Aoun K. 2016. Epidemiological and clinical features of cutaneous leishmaniasis in Kairouan-Tunisia and characteristics in children. Bulletin de la Société de Pathologie Exotique, 109, 80–83. 26850105 10.1007/s13149-016-0475-4

[41] Cissé M, Zida A, Diallo AH, Marty P, Aoun K. 2020. Epidemiology of cutaneous leishmaniasis in West Africa: a systematic review. Bulletin de la Société de Pathologie Exotique, 113, 24–34. 32881442 10.3166/bspe-2020-0115

[42] Croset H, Rioux J-A, Maistre M, Bayar N. 1978. Les Phlébotomes de Tunisie (Diptera, Phlebotomidae). Mise au point systématique, chorologique et éthologique. Annales de Parasitologie Humaine et Comparée, 53, 711–749. 754625

[43] Cross ER, Newcomb WW, Tucker CJ. 1996. Use of weather data and remote sensing to predict the geographic and seasonal distribution of *Phlebotomus papatasi* in southwest Asia. American Journal of Tropical Medicine and Hygiene, 54, 530–536. 8644911 10.4269/ajtmh.1996.54.530

[44] Daly M, Daly S. 2010. Behavior of *Psammomys obesus* (Rodenth: Gerbillinae) in the Algerian Sahara. Zeitschrift für Tierpsychologie, 37, 298–321.

[45] Depaquit J, Lienard E, Verzeaux-Griffon A, Ferté H, Bounamous A, Gantier J-C, Hanafi HA, Jacobson RL, Maroli M, Moin-Vaziri V, Müller F, Özbel Y, Svobodova M, Volf P, Léger N. 2008. Molecular homogeneity in diverse geographical populations of *Phlebotomus papatasi* (Diptera, Psychodidae) inferred from ND4 mtDNA and ITS2 rDNA. Infection, Genetics and Evolution, 8, 159–170. 10.1016/j.meegid.2007.12.001 18243814

[46] Depéret C, Boinet E. 1884. Note sur le microbe du bouton de Gafsa (Tunisie). Bulletin de la Société d’Anthropologie de Lyon, 3, 275–282.

[47] Derbali M, Chelbi I, Ben Hadj Ahmed S, Zhioua E. 2012. *Leishmania major* Yakimoff et Schokhor, 1914 (Kinetoplastida: Trypanosomatidae) in *Meriones shawi* Duvernoy, 1842 (Rodentia: Gerbillidae): persistence of the infection in meriones and its infectivity for the sand fly vector (*Phlebotomus*) *papatasi* Scopoli, 1786 (Diptera: Psychodidae). Bulletin de la Societé de Pathologie Exotique, 105, 399–402. 23055379 10.1007/s13149-012-0259-4

[48] European Centre for Disease Prevention and Control and European Food Safety Authority. 2023. Phlebotomine sandflies maps. Available at: https://ecdc.europa.eu/en/disease-vectors/surveillance-and-disease-data/phlebotomine-maps (accessed 19 April 2026).

[49] Fatemi M, Yaghoobi-Ershadi MR, Mohebali M, Saeidi Z, Veysi A, Gholampour F, Akhondi B, Karimi A, Arandian MH, Amin Mohammadi AM, Rassi Y, Zahraei-Ramazani A, Khamesipour A, Akhavan AA. 2018. The potential role of humans in the transmission cycle of *Leishmania major* (Kinetoplastida: Trypanosomatidae), the causative agent of the old world zoonotic cutaneous leishmaniasis. Journal of Medical Entomology, 55, 1588–1593. 30124876 10.1093/jme/tjy110

[50] Fichet-Calvet E, Jomâa I, Ben Ismail R, Ashford RW. 2003. *Leishmania major* infection in the fat sand rat *Psammomys obesus* in Tunisia: interaction of host and parasite populations. Annals of Tropical Medicine and Parasitology, 97, 593–603. 14511558 10.1179/000349803225001517

[51] Fichet-Calvet E, Jomâa I, Zaafouri B, Ashford RW, Ben-Ismail R, Delattre P. 2000. The spatio-temporal distribution of a rodent reservoir host of cutaneous leishmaniasis. Journal of Applied Ecology, 37, 603–615.

[52] Galati EAB, Rodrigues BL. 2023. A Review of historical Phlebotominae taxonomy (Diptera: Psychodidae). Neotropical Entomology, 52, 539–559. 36897326 10.1007/s13744-023-01030-8

[53] Ghawar W, Toumi A, Snoussi M-A, Chlif S, Zâatour A, Boukthir A, Hamida NBH, Chemkhi J, Diouani MF, Ben-Salah A. 2011. *Leishmania major* infection among *Psammomys obesus* and *Meriones shawi*: reservoirs of zoonotic cutaneous leishmaniasis in Sidi Bouzid (central Tunisia). Vector Borne and Zoonotic Diseases, 11, 1561–1568. 21919726 10.1089/vbz.2011.0712 PMC3263488

[54] Ghawar W, Zaätour W, Chlif S, Bettaieb J, Chelghaf B, Snoussi M-A, Salah AB. 2015. Spatiotemporal dispersal of *Meriones shawi* estimated by radio-telemetry. International Journal of Multidisciplinary Research and Development, 2, 211–216.

[55] Ghrab J, Rhim A, Bach-Hamba D, Chahed MK, Aoun K, Nouira S, Bouratbine A. 2006. Phlebotominae (Diptera: Psychodidae) of human leishmaniosis sites in Tunisia. Parasite, 13, 23–33. 16605064 10.1051/parasite/2006131023

[56] Harrat Z, Pratlong F, Belazzoug S, Dereure J, Deniau M, Rioux JA, Belkaid M, Dedet JP. 1996. *Leishmania infantum* and *L. major* in Algeria. Transactions of the Royal Society of Tropical Medicine and Hygiene, 90, 625–629. 9015497 10.1016/s0035-9203(96)90410-1

[57] Hubálek Z. 2003. Emerging human infectious diseases: Anthroponoses, zoonoses, and sapronoses. Emerging Infectious Diseases, 9, 403–404. 12643844 10.3201/eid0903.020208 PMC2958532

[58] Izri MA, Belazzoug S, Pratlong F, Rioux J-A. 1992. Isolement de *Leishmania major* chez *Phlebotomus papatasi* à Biskra (Algérie). Fin d’une épopée écoépidémiologique. Annales de Parasitologie Humaine et Comparée, 67, 31–32. 1642393 10.1051/parasite/199267131

[59] Jalali H, Enayati AA, Fakhar M, Motevalli-Haghi F, Yazdani Charati J, Dehghan O, Hosseini-Vasoukolaei N. 2021. Reemergence of zoonotic cutaneous leishmaniasis in an endemic focus, northeastern Iran. Parasite Epidemiology and Control, 13, e00206. 33889761 10.1016/j.parepi.2021.e00206 PMC8048008

[60] Jamali H, Bokaie S. 2024. Epidemiological aspects of zoonotic cutaneous leishmaniasis (ZCL) in Iran, the Middle East, and Worldwide: A comprehensive systematic review. medRxiv 2024.2005.2006.24306918.

[61] Jaouadi K, Bettaieb J, Bennour A, Salem S, Ghawar W, Rjeibi MR, Khabouchi N, Gonzalez J-P, Diouani MF, Ben Salah A. 2018. Blood meal analysis of phlebotomine sandflies (Diptera: Psychodidae: Phlebotominae) for *Leishmania spp*. Identification and vertebrate blood origin, Central Tunisia, 2015–2016. American Journal of Tropical Medicine and Hygiene, 98, 146–149. 29165234 10.4269/ajtmh.17-0313 PMC5928704

[62] Karmaoui A. 2020. Seasonal distribution of *Phlebotomus papatasi*, vector of zoonotic cutaneous leishmaniasis. Acta Parasitologica, 65, 585–598. 32347533 10.2478/s11686-020-00201-6

[63] Léger N, Depaquit J. 2001. Les phlébotomes et leur rôle dans la transmission des leishmanioses. Revue Française des Laboratoires, 2001, 41–48.

[64] Lemma W. 2018. Zoonotic leishmaniasis and control in Ethiopia. Asian Pacific Journal of Tropical Medicine, 11, 313. 10.1016/j.apjtm.2017.03.018 28552113

[65] Lewis D, Lane R. 1976. A taxonomic review of *Phlebotomus* (*Idiophlebotomus*) (Psychodidae). Systematic Entomology, 1, 53–60.

[66] Maurelli MP, Zribi L, Fayala NEHB, Manzillo VF, Balestrino I, Hamdi N, Bouratbine A, Gizzarelli M, Rinaldi L, Aoun K, Oliva G. 2024. First detection of *Leishmania major* in dogs living in an endemic area of zoonotic cutaneous leishmaniasis in Tunisia. Parasites & Vectors, 17, 333. 39123245 10.1186/s13071-024-06395-2 PMC11312402

[67] O’Shea B, Rebollar-Tellez E, Ward RD, Hamilton JGC, Naiem D el, Polwart A. 2002. Enhanced sandfly attraction to *Leishmania*-infected hosts. Transactions of the Royal Society of Tropical Medicine and Hygiene, 96, 117–118. 12055795 10.1016/s0035-9203(02)90273-7

[68] Palit A, Bhattacharya SK, Kundu SN. 2005. Host preference of *Phlebotomus argentipes* and *Phlebotomus papatasi* in different biotopes of West Bengal, India. International Journal of Environmental Health Research, 15, 449–454. 16506438 10.1080/09603120500392525

[69] Poulin R. 1995. “Adaptive” changes in the behaviour of parasitized animals: a critical review. International Journal for Parasitology, 25, 1371–1383. 8719948 10.1016/0020-7519(95)00100-x

[70] Rassi Y, Javadian E, Amin M, Rafizadeh S, Vatandoost H, Motazedian H. 2006. *Meriones libycus* is the main reservoir of zoonotic cutaneous leishmaniasis in south Islamic Republic of Iran. Eastern Mediterranean Health Journal, 12, 474–477. 17037718

[71] Ready PD, Croset H. 1980. Diapause and laboratory breeding of *Phlebotomus perniciosus* Newstead and *Phlebotomus ariasi* Tonnoir (Diptera: Psychodidae) from southern France. Bulletin of Entomological Research, 70, 511–523.

[72] Reithinger R, Dujardin J-C, Louzir H, Pirmez C, Alexander B, Brooker S. 2007. Cutaneous leishmaniasis. Lancet Infectious Diseases, 7, 581–596. 17714672 10.1016/S1473-3099(07)70209-8

[73] Rioux JA, Petter F, Akalay O, Lanotte G, Ouazzani A, Seguignes M, Mohcine A. 1982. *Meriones shawi* (Duvernoy, 1842) (Rodentia, Gerbillidae), réservoir de *Leishmania major* Yakimoff et Schokhor, 1914 (Kinetoplastida, Trypanosomatidae) dans le Sud marocain. Comptes Rendus des Séances de l’Académie des Sciences. Série III, Sciences de la Vie, 294, 515–517. 6807510

[74] Rioux JA, Petter F, Zahaf A, Lanotte G, Houin R, Jarry D, Perieres J, Martini A, Sarhani S. 1986. Isolement de *Leishmania major* Yakimoff et Schokhor, 1914 [Kinetoplastida-Trypanosomatidae] chez *Meriones shawi shawi* (Duvernoy, 1842) [Rodentia-Gerbillidae] en Tunisie. Annales de Parasitologie Humaine et Comparée, 61, 139–145. 3729234 10.1051/parasite/1986612139

[75] Rioux JA, Zahaf A, Lanotte G, Dereure J. 1983. *Leishmania major* (Yakimoff et Shokkor, 1914), agent du « clou de Gafsa ». Annales de Parasitologie Humaine et Comparée, 58, 633–634. 6673648 10.1051/parasite/1983586633

[76] Rodhain, F., Perez, C. 1985. Précis d’entomologie médicale et vétérinaire. Notion d’entomologie des maladies à vecteurs. Maloine, Paris. pp. 1–160.

[77] Salah AB, Kamarianakis Y, Chlif S, Ben Alaya N, Prastacos P. 2007. Zoonotic cutaneous leishmaniasis in central Tunisia: Spatio temporal dynamics. International Journal of Epidemiology, 36, 991–1000. 17591639 10.1093/ije/dym125

[78] Sawaf BM el, Mansour NS, Said SM el, Daba S, Youssef FG, Kenawy MA, Beier JC. 1989. Feeding patterns of *Phlebotomus papatasi* and *Phlebotomus langeroni* (Diptera: Psychodidae) in El Agamy, Egypt, Journal of Medical Entomology, 26, 497–498. 2795623 10.1093/jmedent/26.5.497

[79] Service MW, editor. 2022. Encyclopedia of arthropod-transmitted infections of man and domesticated animals. Wallingford: CABI Publishing.

[80] Shahabi S, Azizi K, Asgari Q, Sarkari B. 2024. *Leishmania major* infection in synanthropic rodents: evidence for the urbanization of zoonotic cutaneous leishmaniasis (ZCL) in southern Iran. Canadian Journal of Infectious Diseases and Medical Microbiology, 2024, 4896873. 38487175 10.1155/2024/4896873 PMC10940013

[81] Takahashi EA, Masoud L, Mukbel R, Guitian J, Stevens KB. 2020. Modelling habitat suitability in Jordan for the cutaneous leishmaniasis vector (*Phlebotomus papatasi*) using multicriteria decision analysis. PLOS Neglected Tropical Diseases, 14, e0008852. 33226979 10.1371/journal.pntd.0008852 PMC7721129

[82] Theodor O. 1948. Classification of the Old World species of the subfamily phlebotominae (Diptera, Psychodidae). Bulletin of Entomological Research, 39, 85–115. 18865548 10.1017/s0007485300024305

[83] Théodoridès J. 1997. Note historique sur la découverte de la transmission de la leishmaniose cutanée par les phlébotomes. Bulletin de la Societé de Pathologie Exotique, 90, 177–178. 9410253

[84] World Health Organization. 2023. Leishmaniasis. WHO Fact sheet. Available at: https://www.who.int/news-room/fact-sheets/detail/leishmaniasis (accessed 24 February 2026).

[85] Yaghoobi-Ershadi MR, Akhavan AA, Zahraei-Ramazani AR, Jalali-Zand AR, Piazak N. 2005. Bionomics of *Phlebotomus papatasi* (Diptera: Psychodidae) in an endemic focus of zoonotic cutaneous leishmaniasis in central Iran. Journal of Vector Ecology, 30, 115–118. 16007964

